# Sphingolipid metabolism and drug resistance in ovarian cancer

**DOI:** 10.20517/cdr.2018.06

**Published:** 2018-09-19

**Authors:** Kelly M. Kreitzburg, Robert C. A. M. van Waardenburg, Karina J. Yoon

**Affiliations:** Department of Pharmacology and Toxicology, University of Alabama at Birmingham, Birmingham, AL 35294, USA.

**Keywords:** Ovarian cancer, drug-resistance, ceramide, sphingosine-1-phosphate, sphingolipid metabolism

## Abstract

Despite progress in understanding molecular aberrations that contribute to the development and progression of ovarian cancer, virtually all patients succumb to drug resistant disease at relapse. Emerging data implicate bioactive sphingolipids and regulation of sphingolipid metabolism as components of response to chemotherapy or development of resistance. Increases in cytosolic ceramide induce apoptosis in response to therapy with multiple classes of chemotherapeutic agents. Aberrations in sphingolipid metabolism that accelerate the catabolism of ceramide or that prevent the production and accumulation of ceramide contribute to resistance to standard of care platinum- and taxane-based agents. The aim of this review is to highlight current literature and research investigating the influence of the sphingolipids and enzymes that comprise the sphingosine-1-phosphate pathway on the progression of ovarian cancer. The focus of the review is on the utility of sphingolipid-centric therapeutics as a mechanism to circumvent drug resistance in this tumor type.

## INTRODUCTION

Ovarian cancer is the fifth leading cause of cancer-related deaths and the leading cause of death among women with gynecological malignancies. Although progress has been made in understanding the biology of ovarian cancer, progress in treating patients with this tumor type has been equivocal. Fewer than half of patients achieve complete remission^[1]^. Seventy-five percent of women are diagnosed with advanced, metastatic disease mainly due to limitations in early detection and nonspecific symptoms. While a majority of women respond favorably to tumor debulking and frontline chemotherapy with carboplatin and paclitaxel, more than half will relapse within 18 months of diagnosis with drug resistant disease^[2]^.

Development of chemotherapy resistant disease is a fundamental obstacle in treating many tumor types and is a factor in treatment failure for many patients with advanced, disseminated disease. Due to the differences in molecular characteristics and complexities among tumor types, the mechanisms of and pathways involved in the emergence of a chemoresistant phenotype may be unique to specific classes of agents and tumor types. However, general resistance mechanisms include increased drug efflux, enhanced DNA damage response, defective apoptotic signaling, or activation of anti-apoptotic proteins^[3]^. Acquired resistance may include drugs with different mechanisms of action, to which tumors have not yet been exposed. Due to the late stage at which most ovarian cancers are diagnosed, a high proportion of tumors are resistant to platinum-based therapy at diagnosis. Because few alternative therapies are available, the 5-year survival for these patients remains at less than 50%. New treatment strategies are needed.

Manipulation of the sphingolipid-mediated sphingosine-1-phosphate (S1P) pathway may represent such a strategy. The S1P pathway, including sphingolipid metabolites, regulates multiple cellular processes including proliferation, neovascularization, migration, invasion, and metastasis by controlling cell signal transductions networks that contribute to both tumorigenesis and tumor progression^[4–6]^. Sphingolipids also contribute to the structural integrity and fluidity characteristics of cell membranes^[6,7]^. Few therapeutic agents directly target S1P pathway proteins, but this pathway can influence the efficacy of several classes of chemotherapeutic agents, including docetaxel, doxorubicin and cyclophosphamide. Further, aberrations in sphingolipid metabolism are associated with chemoresistance^[8–11]^. This review details current understanding of how the S1P pathway impacts the development and progression of ovarian cancer and addresses the therapeutic implications of targeting this pathway in this tumor type. The focus of the review is on one of the core lipids of the S1P pathway, ceramide, and on the role of ceramide in inducing tumor cell death.

## THE SPHINGOLIPID RHEOSTAT AND CANCER

**Figure 1.**
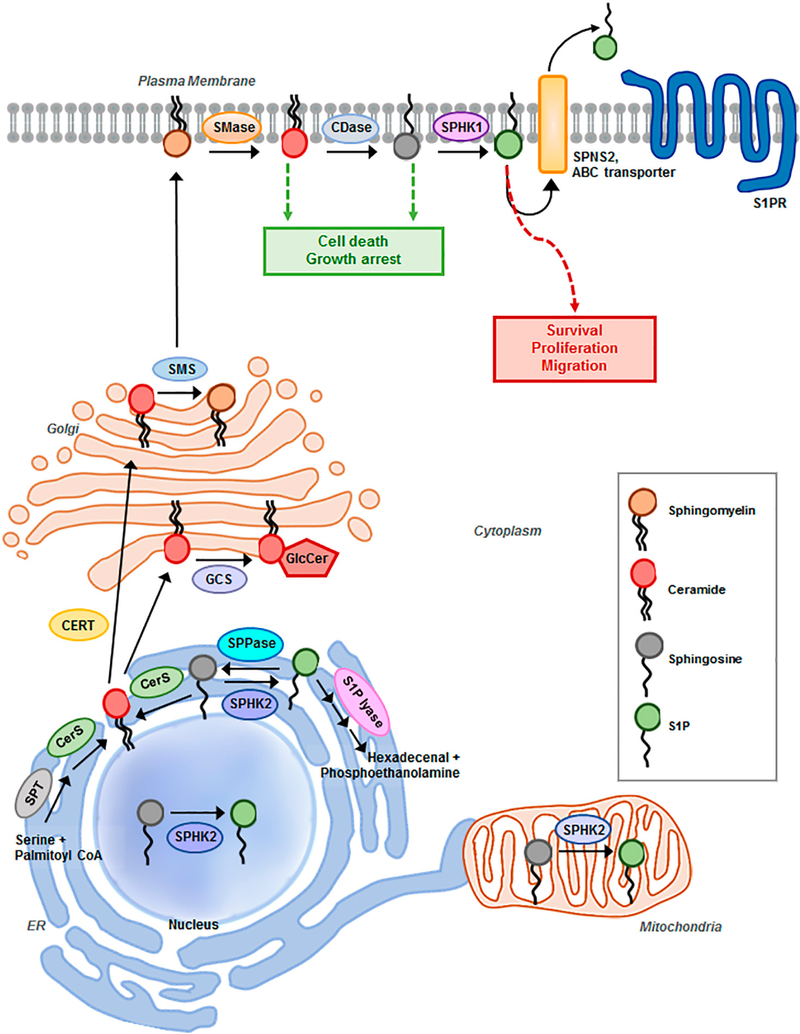
Schematic representation of sphingosine-1-phosphate (S1P) pathway. Summary of sphingolipid degradation and synthesis and major components of the S1P metabolic pathway implicated in ovarian cancer progression and drug resistance. SMase: sphingomyelinase; CDase: ceramidase; SPHK1/2: sphingosine kinase 1/2; SPNS2: Spinster homolog 2; ABC transporter: ATP-binding cassette transporters, ABCA1, ABCC1, and ABCG1; S1PR1,2,3,4,5: S1P receptor; SMS: sphingomyelin synthase; GCS: glucosylceramide synthase; GlcCer: glucosylceramide; CERT: ceramide transport protein; SPT: serine palmitoyltransferase; CerS: ceramide synthase; SPPase: S1P Phosphatases

S1P pathway signaling is regulated primarily by the relative levels of ceramide, sphingosine, and S1P, which comprise the three core lipids of the S1P pathway. These lipids play critical roles in cellular processes such as cell growth, differentiation, death, and motility^[12]^ [Figure 1]. Mechanisms that regulate the synthesis, catabolism, and clearance of these bioactive sphingolipids are tightly coupled to specific stimuli that engage and regulate downstream effectors, distinguishing them from lipids that have predominantly structural functions^[4,8]^. Adding another layer of complexity, sphingolipid metabolism constitutes an interconnected network comprised of numerous pathways that not only regulate the levels of individual bioactive sphingolipids, but also their interconversion and the sphingolipid balance^[8]^. The concept that the relative levels of the three core lipids are tightly regulated to influence cellular processes is referred to as the ceramide-sphingosine-S1P rheostat model^[13]^. S1P is mitogenic and promotes growth, motility, and angiogenesis, whereas the S1P precursors sphingosine and ceramide mediate anti-proliferative and cytotoxic stress responses including apoptosis, cell cycle arrest, lethal autophagy, and growth suppression *in vitro* and *in vivo*^[6,13–15]^.

These three core sphingolipid metabolites are rapidly interconverted in response to various stimuli such as growth factors, inflammatory stimuli and stress. The resulting changes in relative levels of these lipids, in turn, mediate specific responses^[6,15]^. For example, cellular stress such as chemotherapy, radiation, or oxidative stress increases levels of ceramide and sphingosine through the activation of *de novo* synthesis, sphingomyelin hydrolysis, or the salvage pathway which recycles sphingosine to promote apoptosis. However, tumor cells often have relatively low levels of ceramide, due to increased activities of ceramide metabolizing enzymes such as glucosylceramide synthase (GCS), sphingomyelin synthase (SMS), ceramide kinase (CERK), acid ceramidase (AC), or sphingosine kinase (SPHK). These enzymes convert ceramide to glucosylceramide (GlcCer), sphingomyelin, ceramide-1-phosphate (C1P), or S1P sphingolipids, respectively, each of which has pro-survival activity^[7,11,12]^. In normal cells, the tightly regulated balance of synthesis and degradation of sphingosine, ceramide and S1P maintains sphingosine and S1P levels ~10-fold lower than the level of ceramide^[16]^. Of note, hydrolysis of less than 3% of ceramide can exponentially increase sphingosine and subsequent S1P levels^[12]^. The interdependent nature of sphingolipid metabolism facilitates rapid interconversion among core lipids, to orchestrate diverse cellular responses^[5,15]^. The complex regulation of the S1P pathway varies with cell type, and the abundance or deficiency of sphingolipids and their respective metabolizing enzymes make it challenging to define the mechanisms that regulate initiation or progression of a particular tumor type^[4,17]^. This review focuses on the function and potential utility of the S1P pathway as a therapeutic target in ovarian cancer.

## SPHINGOLIPID METABOLISM

S1P synthesis is tightly regulated by the metabolism of ceramide. Ceramide sits at the hub of sphingolipid metabolism as the neutral, lipid building block for complex sphingolipids and glycosphingolipids, serving as a substrate for more than 11 different enzymes^[12]^ [Figure 1]. Ceramide biosynthesis occurs either from the breakdown of membrane-resident sphingomyelin by sphingomyelinases or *de novo* from the condensation of serine and palmitoyl-CoA, catalyzed by serine palmitoyltransferase (SPT) to form 3-ketosphinganine. Subsequently, it is reduced to sphinganine by 3-ketosphinganine reductase and synthesized into dihydroceramide by (dihydro) ceramide synthases. Referred to as ceramide synthases (CERS1–6), these are the rate-limiting enzymes for the synthesis of endogenous ceramide, with different fatty acyl-CoA substrate preferences. Various chain-length ceramides are ultimately generated as the product of dihydroceramide desaturase^[12,18]^. The *de novo* formation of ceramide is induced by several stress-factors including tumor necrosis factor-α, hypoxia, and chemotherapeutic agents^[19]^. Ceramide can be converted to sphingomyelin by sphingomyelin synthase, glycosylated by glucosylceramide synthase (GCS) to form glucosylceramide, or ceramide can be hydrolyzed to form sphingosine by ceramidases (acid, neutral, or alkaline), characterized by the pH required for optimal enzymatic activity^[7,20]^. Ultimately, sphingosine is phosphorylated by sphingosine kinase isozymes (SPHK1 or SPHK2) to generate the bioactive lysophospholipid S1P. Through a range of different pathways, S1P acts as both an extracellular and intracellular signaling molecule. S1P can be transported out of the cell by members of the ABC transporter family and by spinster homolog 2 (Spns2), where it exists in high nanomolar concentrations in the blood^[21]^. Upon exit from the cell, S1P engages with 5 specific G-protein coupled receptors (GPCRs), referred to as S1PR1–5, in an autocrine or paracrine manner to induce downstream signal transduction cascades that promote proliferation and migration^[11,16]^. Alternatively, S1P can be rapidly metabolized by S1P phosphatases to reform sphingosine or irreversibly cleaved by S1P lyase (SGPL1) to yield phosphoethanolamine and hexadecanol, the final step in sphingolipid degradation^[7,12,16]^. Sustaining the flux between S1P generation and degradation is critical in regulating the balance of sphingolipids and plays a key role in pathological processes of tumorigenesis^[17,22]^. Specifically, with respect to ovarian cancer, S1P has been indicated to contribute to metastatic potential by stimulating the migration, chemotaxis, and invasion of ovarian cancer cells in several studies^[23–26]^. S1P may inhibit or enhance migration and invasion in a cell-type- and concentration-dependent manner^[25]^.

## SPHINGOLIPIDS IN OVARIAN CANCER AND DRUG RESISTANCE

Several literature have implicated dysregulated sphingolipid metabolism as key contributor of the progression and resistance of ovarian cancer. Using RNA-seq to compare and identify transcriptional variants between matched pairs of carboplatin and paclitaxel-treated *vs*. control patient-derived xenograft (PDX) models of ovarian cancer, Dobbin and colleagues identified S1P signaling in the top three most transcriptionally altered pathways following chemotherapy treatment^[27]^. Sphingolipid metabolizing enzymes directly involved in regulating the ceramide-sphingosine-S1P rheostat play a crucial role in cell survival and have been directly correlated with drug resistance in ovarian cancer^[20,28]^. Specifically, increased expressions of ceramide transport protein (CERT), SPHK1, SPHK2, and glucosylceramide synthase (GCS) have been associated with resistance to paclitaxel, doxorubicin, and N-(4-hydroxylphenyl) retinamide (fenretinide) chemotherapies and apoptotic responses^[29–34]^.

Because altered levels or activity of bioactive sphingolipids regulate biological processes that influence tumor progression, several laboratories have investigated approaches to increase levels of the proapoptotic lipid ceramide and to decrease levels of the antiapoptotic lipid S1P^[7,13,15]^. With respect to ovarian cancer, the higher levels of S1P present in the ascites fluid of ovarian cancer patients skew the ratio of the three core lipids, to promote proliferation, angiogenic potential, and dissemination of ovarian tumors^[11,35]^. Further, increased levels of sphingomyelin, glucosylceramide, and galactosylceramide have been postulated to confer a multidrug resistant phenotype in ovarian cancer cells^[29,36,37]^. Consistent with these observations, the level of ceramide is lower in ovarian tumor cells than in normal ovarian tissue and is further attenuated in paclitaxel-resistant compared to paclitaxel-sensitive ovarian cancer cells, again skewing the ratio of ceramide:S1P in favor of the anti-apoptotic lipid S1P in tumor cells^[8,38]^. These studies suggest that the balance between ceramide and S1P levels is critical in mediating drug-sensitivity and survival of tumor cells, which underscore targeting the rheostat for the evaluation of rational anticancer regimens.

## PUTATIVE MECHANISMS BY WHICH CORE LIPIDS REGULATE APOPTOSIS

While development of such therapies may be challenging because of the rapid interconversion among core lipid components, it is well documented that increases in proapoptotic ceramide occur through a variety of mechanisms: (1) *de novo* synthesis, the conversion of serine and palmitoyl-CoA to ceramide via multiple steps; (2) hydrolysis of sphingomyelin; (3) inhibition of ceramide hydrolysis; and (4) hydrolysis of glucosylceramide (or inhibition of glucosylceramide)^[15]^. Induction of apoptosis by ceramide also occurs through multiple mechanisms: first, apoptosis through mitochondrial activation by forming ceramide platforms on cell membranes which subsequently invaginate and fuse with the mitochondria; also known as “the kiss of death”, ultimately leading to the induction of apoptosis. Second, ceramide can also form channels in mitochondrial membranes, to induce mitochondrial outer membrane permeabilization (MOMP). MOMP, in turn, promotes the release of apoptotic proteins such as cytochrome c and low molecular weight intermembrane space proteins into the cytoplasm^[15]^.

Radiation-induced ceramide accumulation has been shown to function as a second messenger to activate the intrinsic apoptosis pathway and induce senescence through inhibition of telomerase activity, an enzyme overexpressed in approximately 90% cancer cells, enabling cells to escape senescence and acquire immortality^[8,11,15]^. Recently, El Kaffas *et al*.^[39]^ demonstrated that activation of the acid sphingomyelinase-ceramide pathway is necessary for radiosensitization following ultrasound-stimulated microbubble (USMB) exposure. This study is the first to investigate and highlight the role of acid sphingomyelinase-ceramide signaling in USMB-mechanotransducive vascular therapy, showing minimal tumor cell death and responses in S1P-treated and acid-sphingomyelinase knockout mice compared to wild-type mice implanted with fibrosarcoma xenografts. Approaches to increase ceramide levels merit investigation.

Alternatively, approaches to decrease the anti-apoptotic lipid S1P^[7,11,13]^ may also be useful therapeutically. S1P blocks apoptosis by stabilizing mitochondrial membrane potential, thus preventing cytochrome c release from the mitochondria^[40]^. Specifically, with respect to ovarian cancer, S1P has been indicated to contribute to metastatic potential by stimulating the migration, chemotaxis, and invasion of ovarian cancer cells in several studies^[23–26]^. S1P may inhibit or enhance migration and invasion in a cell-type- and concentration-dependent manner^[25]^. The antiapoptotic activity of S1P can be influenced by the level of S1P receptor expression, character of preexisting stress fibers, and levels of enzymes involved in extracellular matrix (ECM) remodeling and invasion^[24,41]^.

Angiogenesis supports invasion and metastasis of solid tumors. S1P induces expression and/or secretion of several pro-angiogenic cytokines such as VEGF, IL-8, and IL-6, to promote vascular network formation^[42,43]^. Further the expression of SPHK1 and S1PR1/3 was correlated with microvascular density of ovarian cancer tissue, and inhibition of SPHK1 or S1PR1/3 attenuated angiogenic potential and angiogenic factor secretion of ovarian cancer cells *in vitro* and *in vivo*^[42]^.

Thus, therapeutic approaches would aim to promote ceramide accumulation and suppress S1P accumulation, to inhibit tumor growth and overcome drug resistance.

## SPHK1 AND SPHK2

Despite their metabolic redundancy for generating S1P, SPHK1 and SPHK2 possess distinct, cell type-dependent characteristics, with differences in level of expression and intracellular localization^[11,44,45]^. SPHK1 localizes primarily in cytosol and cell membrane, whereas SPHK2 localizes at the nucleus, mitochondria and endoplasmic reticulum (ER). These distinct subcellular distributions have been cited as the factors that determine the divergent biochemical roles of SPHK1 and SPHK2^[45,46]^. Of the two, SPHK1 is better characterized and high levels of expression of this enzyme have been shown to promote oncogenic transformation, tumor growth, and drug resistance in ovarian cancer cells^[29,47–49]^. The oncogenic signaling mediated by SPHK1 depends on its activation, translocation to the plasma membrane, and subsequent catalysis of sphingosine to S1P^[13,50]^. SPHK1 activity and expression are augmented by a range of agonists including protein kinase activators, tyrosine kinase growth factors, GPCR ligands, small GTPases, proinflammatory cytokines, and calcium^[11]^. Interestingly, p53 activation in response to DNA damage can mediate proteolytic cleavage and inactivation of SPHK1, to promote the initiation of apoptosis^[51]^. mRNA and protein levels of SPHK1 are higher in primary ovarian tumors compared to their non-cancerous tissues and are associated with reduced 5-year survival^[52–54]^. Elevated SPHK1 expression accelerates the conversion of ceramide to S1P, while removing ceramide from the biosynthetic pool via dihydrosphingosine phosphorylation; thus, playing a role in regulating cellular ceramide levels^[8,53]^. In a recent study, Lee *et al*.^[29]^ examined the antiproliferative effect of siRNA targeting SPHK1 combined with the sphingosine analog FTY720 in cultured EOC cell lines, and in xenografts and a patient-derived xenograft (PDX) model of clear cell carcinoma (CCC) in mice. SPHK1-siRNA plus FTY720 inhibited proliferation and invasion, and increased apoptosis in chemotherapy-resistant as well as -sensitive models of EOC *in vitro*. Furthermore, treatment with FTY720 *in vivo* inhibited tumor growth and proliferation (*P* < 0.05) in cell line-derived xenografts models and a PDX model of CCC^[29]^. These data support the hypothesis that targeting SPHK1 has a therapeutic potential in ovarian cancer. In another study, SPHK1 was shown to be highly expressed in the tumor stroma of HGSOC and required for the differentiation and tumor promoting function of cancer-associated fibroblasts^[55]^.

Compared to the extensively investigated SPHK1 isoform, the functions and mechanisms of SPHK2 in cancer remain largely elusive and the roles of SPHK2 in cancer cells are not fully understood with inconsistencies in published data^[44,56]^. For example, Liu *et al*.^[57]^ revealed SPHK2 contains a putative BH3 motif, which is essential in the activation and initiation of apoptosis by BH3-only proteins, and mutations in the highly conserved catalytic domain decreased its ability to induce apoptosis in NIH 3T3 fibroblasts, human embryonic kidney (HEK293), PC12 pheochromocytoma, and MCF-7 breast cancer cells. In contrast, work by Gao and Smith^[58]^ demonstrated that SPHK2 contributed to proliferation and survival of breast adenocarcinoma cells and kidney clear cell carcinoma and adenocarcinoma cells *in vitro*. Additional studies conducted using SPHK2-siRNA indicate that decreased expression of SPHK2 enhanced apoptosis and decreased resistance to etoposide and doxorubicin in cell lines derived from lung, breast, and colon tumors^[59,60]^. Further, the specific localization of SPHKs also contributes to the cell function. Nuclear localized SPHK2 and S1P have been reported to have anti-proliferative roles; through which SPHK2 forms a repressor complex with histone H3 and histone deacetylase 1 and 2 (HDAC1/2) producing S1P that regulates of histone acetylation, as a part of epigenetic regulation of gene expression^[13,50,61]^. Alternatively, nuclear SPHK2-derived S1P has been shown to bind hTERT and allosterically mimic protein phosphorylation which limits proteasomal degradation and maintains telomere integrity and stabilization, thereby, bypassing replicative senescence and enhancing tumor growth^[44]^. Data for the function of SPHK2 in ovarian cancer cells are limited. Dai *et al*.^[42]^ showed that SPHK1, but not SPHK2, expression was correlated with microvascular density (MVD) of ovarian cancer cells and that the angiogenic factor secretion by ovarian cancer cells could be attenuated by SPHK1, but not SPHK2 inhibition and subsequently restored upon addition of S1P. Alternatively, few stimuli have been shown to induce SPHK2-mediated S1P formation, such as epidermal growth factor (EGF), PMA, TGFβ, and FcεRI triggering^[29]^.

## CERAMIDE TRANSPORT AND METABOLISM

*De novo* ceramide biosynthesis pathway is initiated at the cytosolic leaflet of the endoplasmic reticulum (ER), where the enzymes required for ceramide synthesis localize. Ceramide is subsequently transported to vesicular or non-vesicular loci^[19]^. Ceramide either undergoes vesicular trafficking to the cis-Golgi where it is converted to glucosylceramide (GlcCer) or gets transported to the trans-Golgi where it is preferentially incorporated into sphingomyelin^[20,62]^. The ceramide transfer protein CERT, encoded by the COL4A3BP gene, regulates this non-vesicular transport, to control the conversion of ceramide to sphingomyelin by sphingomyelin synthase (SMS)^[7,19,63,64]^. siRNA-mediated silencing of COL4ABP sensitizes diverse cells types, including ovarian, colorectal, and HER2-positive breast cancer cells to doxorubicin, cisplatin, 5-FU, and paclitaxel. The mechanism by which this sensitization occurs is thought to be through the induction of ceramide-mediated ER stress or lysosome-associated membrane glycoprotein 2 (LAMP2)-dependent autophagic flux. Consistent with this hypothesis, drug-resistant SKOV3-TR ovarian cancer and ADR/RES breast cancer cells express relatively high levels of CERT, and silencing of COL4ABP sensitizes these cell lines to paclitaxel-induced cell death^[7,28,32]^. Thus, inhibition of ceramide metabolism via targeting CERT-mediated trafficking of ceramide as well as conversion into glycosphingolipids may provide a novel strategy for sensitizing ovarian cancer cells to several classes of chemotherapeutic agents.

Glucosylceramide synthase (GCS) transfers glucose from UDP-glucose to ceramide to form glucosylceramide, the precursor for approximately 90% of mammalian glycosphingolipids (GSLs). Both ceramide and GSLs play critical roles in modulating cellular signaling and gene expression, and thus modulating tumorigenesis, cancer progression, and the efficacies of cancer therapies^[65,66]^. Ceramide glycosylation by GCS is the rate-limiting step in glycosphingolipid synthesis and is essential in regulating the balance between apoptotic ceramide and antiapoptotic glucosylceramide^[20]^. Comparison of GSL expressions using matrix-assisted laser desorption/ionization-mass spectroscopy (MALDI-MS) and MALDI-MS/MS showed increased and differential glycosylation of GSLs in the epithelial ovarian cancer SKOV3 cell line compared to the nontumorigenic epithelial ovarian T29 cell line, with five neutral globo-series GSLs detected only in the SKOV3 cell line^[66]^. Several studies have highlighted the influence of ceramide and glycolipid metabolism on function and expression of genes involved in response and metabolism of chemotherapies such as cisplatin, doxorubicin, vinblastine, paclitaxel and inflammatory responses to physiological stimuli such as tumor necrosis factor-α and cyclooxygenase-2^[34,65,67–71]^. In mechanistic studies associated with clinical trials, overexpression of GCS has been associated with poor prognosis and multidrug resistance in several tumor types including ovarian, breast, and colorectal cancers. These observations suggest that high levels of GCS expression merits investigation as a biomarker of clinical response or tumor progression^[20,70,72,73]^. Therefore, targeting the metabolism (and glycosylation) of ceramide presents an effective strategy for anticancer drug development to potentiate cellular sensitivity to ceramide-induced cell death and chemotherapeutics. These studies underscore ceramide’s essential role in mediating signaling cascades in response to cellular stressors such as physiological stimuli, chemotherapy, and ionizing radiation and provide rationale to investigate therapeutic strategies that target the metabolism (and glycosylation) of ceramide as anticancer treatments to potentiate cellular sensitivity to ceramide-induced cell death.

Interestingly, GCS overexpression is sometimes coincidental with overexpression of the multidrug resistance 1 gene (MDR1) in drug-resistant breast, ovary, cervical and colon cancer cells^[69]^. MDR1 encodes the drug efflux transporter P-glycoprotein (P-gp), which facilitates export of several classes of chemotherapeutic agents including Vinca alkaloids, anthracyclines, paclitaxel, actinomycin D, and epipodophyllotoxins^[68]^. Furthermore, Liu *et al*.^[34]^ demonstrated that suppression of GCS enhanced sensitivity to doxorubicin and restored ceramide-mediated, p53-dependent apoptosis *in vitro* and *in vivo* in p53 mutant OVCAR-8, NCI/ADR-RES, and A2780ADR ovarian cancer cells. Mechanistically, GCS suppression increased long chain C18- and C24-ceramide species which, in turn, modulate pre-mRNA splicing to restore wild-type p53 expression^[74]^. However, in another cell type, inhibition of GCS expression resulted in cytokinetic dysfunction and multinucleation of human cervical adenocarcinoma (HeLa) cells, which have been associated with chemoresistance^[75]^. Consequently, while inhibition of GCS, to prevent the metabolism of ceramide to glucosylceramide, may provide an effective means to circumvent drug resistance, the effects of inhibiting GCS may be cell type-dependent and the utility of this approach needs additional studies before inhibition of GCS is considered a useful therapeutic approach.

## SPHINGOLIPID-BASED ANTICANCER THERAPEUTICS

As discussed above, sphingolipids play a regulatory role in determining cell fate. Multiple approaches have been investigated for influencing sphingolipid metabolism, to overcome drug resistance in ovarian cancer cells. Approaches that have demonstrated efficacy in ovarian cancer models include the use of synthetic ceramide analogs, inhibitors of SPHK, neutralization of secreted S1P, and S1PR antagonists. Studies addressing each approach are summarized below and tabulated in Table 1.

## CERAMIDE ANALOGS

Cell-permeable ceramide analogs or mimetics induce apoptosis in cancer cells^[76]^. The solubility and bioavailability of such analogs has been increased by replacing the long-chain fatty acid of endogenous ceramide with short chain fatty acids (C2-, C6-, or C8-), or by encapsulating ceramide in liposomes or polymeric nanoparticles^[9]^. As a single “agent” bioavailable ceramide increases intracellular ceramide levels and induces apoptosis. Even greater efficacy has been reported when ceramide analogs or formulations are combined with more conventional chemotherapeutic agents^[77–79]^. As a first example, CAOV3 ovarian cancer cells exposed to C6-ceramide and paclitaxel demonstrated high levels of endocytic vesicle formation and synergy in inhibiting cell proliferation and migration^[37,80]^. A second example, the combination of paclitaxel with C6-ceramide-encapsulated in poly(ethylene oxide)-modified poly(epsilon-caprolactone) (PEO-PCL) nanoparticles restored sensitivity of taxane-resistant SKOV3.TR ovarian cancer cells to paclitaxel^[77]^. A third example, Zhu *et al*.^[81]^ demonstrated that C6-ceramide and the histone deacetylase inhibitor (HDACI) trichostatin A (TSA) were synergistic in models of ovarian and pancreatic cancer *in vitro* and *in vivo*. The synergistic effects of this combination were attributed to increases in α-tubulin hyperacetylation and intracellular ceramide accumulation, the release and activation of protein phosphatase 1 (PP1), and subsequent dephosphorylation of AKT^[81]^.

Preclinical data that support the use of ceramide nanoliposomes (CNL) are available for several preclinical tumor models of hepatocellular carcinoma, breast, melanoma, and ovarian cancers, and leukemia models^[77,82–91]^. These studies provided strong support for the FDA phase I first-in-man-dose-escalation study in patients with advanced solid tumors (NCT02834611)^[82,92,93]^. Mechanistically, using SKOV3 ovarian cancer cells, Zhang *et al*.^[82]^ made the novel observation that CNL targets the pseudokinase mixed lineage kinase like (MLKL) domain to induce necroptosis *in vitro* and *in vivo*. Their findings demonstrated an inverse relationship between monomeric MLKL expression and CNL efficacy and suggest that MLKL expression may serve as a biomarker of therapeutic efficacy of CNL-based therapy^[82]^. Also, Kitatani *et al*.^[94]^ demonstrated that C6-ceramide liposomes suppressed ovarian cancer cell motility *in vitro* and inhibited peritoneal metastasis in a murine xenograft model. The study by Kitatani *et al*.^[94]^ also showed that C6-ceramide liposomes suppressed ovarian cancer cell motility *in vitro* and inhibited peritoneal metastasis in a murine xenograft model *in vivo*. Furthermore, metastasis of PI3KC2β knocked-down xenografts were insensitive to treatment with ceramide liposomes, suggesting the role of ceramide as a metastasis-suppressor lipid and an involvement of ceramide interaction with PI3KC2β in metastasis suppression^[94]^.

## S1P-SPECIFIC ANTIBODIES

S1P-specific murine (LT1002, Sphingomab) and humanized (LT1009, Sonepcizumab) monoclonal antibodies bind and neutralize S1P, and inhibit the activity of the endogenous enzyme by lowering circulating S1P. Although inactive, S1P-Ab complexes bind to the S1P receptor (S1PR) and competitively inhibit the binding of the active endogenous enzyme to this receptor, thereby decreasing the S1P pathway function. Anti-S1P antibodies reduced the expression and activity of hypoxia-inducible factor 1α (HIF-1α), secretion of the angiogenic factors IL-6, IL-8, vascular endothelial growth factor (VEGF), and basic fibroblastic growth factor (bFGF) and decreased vessel formation in *in vitro* and *in vivo* models of ovarian, breast, prostate and lung cancers^[95–97]^. The antiangiogenic effect of S1P antagonism in preclinical models led to the evaluation of Sonepcizumab in a phase II clinical trial in patients with metastatic renal cell carcinoma (mRCC) who were refractory to anti-VEGF therapy^[98,99]^. Forty patients who had undergone a median of three prior regimens were enrolled. Patients achieved a median overall survival of 21.7 months was observed, but the study did not achieve its primary endpoint of a 2-month progression-free survival. While Sonepcizumab demonstrated an encouraging overall survival of > 20 months in a heavily pretreated population of patients with mRCC, only 10% (4 patients) demonstrated a partial response, with a median duration of response of 5.9 months. Interestingly, biomarker studies showed simultaneous increases in serum S1P and antibody concentrations, but no significant association was found between response to therapy and increases in S1P levels.

## FTY720

FTY720 (Fingolimod) is an FDA-approved, first-line, immunomodulatory therapy for relapsing multiple sclerosis, an inflammatory disorder of the central immune system. FTY720 is a sphingosine analog derived from the potent serine palmitoyltransfease (SPT) inhibitor myriocin, and is a prodrug phosphorylated primarily by SPHK2 to generate P-FTY720 which is a structural analog of S1P. FTY720 functions as an antagonist of S1PR1, thereby sequestering circulating lymphocytes in lymphoid tissues^[100–103]^. In addition to its primary indication as an S1PR ligand and immunosuppressive role, FTY720 has shown antitumor efficacy in multiple *in vitro* and *in vivo* models. FTY720 impacts multiple cell functions and pathways including motility, proliferation, death, angiogenesis, inflammation, and S1P^[104–106]^. FTY720 has been demonstrated as a competitive inhibitor (with sphingosine) of SPHK1 with a Kic of 2 μmol/L^[107,108]^ and destabilizes SPHK1 by facilitating SPHK1 degradation via ubiquitination in human pulmonary artery smooth muscle, breast cancer, and androgen-independent prostate cancer cells^[108,109]^. Although somewhat controversial, compelling evidence suggests that the anticancer effects of FTY720 are independent of phosphorylation and that the “prodrug” FTY720 is an active antitumor agent^[104,110]^. Due to differences in the expression levels and tissue distributions of SPHK2, FTY720-P phosphatases, and the ATP-binding cassette (ABC) transporters ABCA1, ABCB1, ABCC1, and ABCG2, as well as the multipass transmembrane family protein SPNS2, the concentration of FTY720-P differs between cells and tissues. Therefore, even when the intended use of FTY720 is as an antitumor agent, relatively high levels of FTY720-P are likely to be present in lymphoid tissue and to exert a potent immunosuppressive effect^[109,111,112]^.

Notably, tumor cells resistant to radiation and conventional chemotherapeutic agents such as cisplatin, topotecan, doxorubicin, etoposide, and tamoxifen are sensitive to FTY720 as a single agent and show additive or synergistic effects when combined with chemotherapy or radiation. FTY720 has been shown to potentiate the effects of these agents in models of ovarian, glioblastoma, prostate, breast, colon, melanoma and pancreatic cancers^[61,104,113–116]^. These preclinical data support the use of FTY720 and this agent is currently being evaluated in combination with radiation and temozolomide in a phase I clinical trial in newly diagnosed high grade glioma patients (NCT02490930)^[117]^. FTY720 is toxic to ovarian cancer cells independent of their sensitivity to cisplatin, carboplatin, and paclitaxel (Kreitzburg and Yoon, unpublished data) and can initiate both autophagic and necrotic death and apoptosis^[29,104,106]^. In ovarian cancer cell lines, FTY720 inhibited SPHK1 activity, angiogenesis, invasion, and proliferation. Furthermore, administration of FTY720 to mice bearing cell line xenograft and PDX models of ovarian cancer inhibited tumor growth^[29]^.

## SPHK INHIBITORS

Because S1P contributes to cancer progression and drug resistance, the SPHK enzymes that generate S1P are also potentially useful targets for cancer therapy. Safingol (L-threo-dihydrosphingosine), a synthetic isomer of sphinganine, is the first molecule designed to inhibit SPHK1/2 to be evaluated in clinical trial. Safingol functions as a competitive inhibitor of SPHK1/2 and an inhibitor of ceramide kinase to increase ceramide levels. This compound also appears to inhibit protein kinase C, by an unknown mechanism^[118,119]^. *In vitro* data demonstrate that combinations of safingol with agents such as doxorubicin, cisplatin, or mitomycin C are synergistic in models of ovarian, colon, breast, cervical, and head and neck squamous cell cancer models. These combinations increase apoptosis and lethal autophagy induced by these conventional drugs as single agents. Based on *in vitro* data, safingol was combined with cisplatin for treatment of patients with advanced solid tumors in trial NCT0084812. Although safingol possesses limited activity as a single-agent *in vivo*, it potentiates the efficacy of cisplatin with little or no increase in toxicity and is being further evaluated in a phase I clinical trial in combination with fenretinide in patients with relapsed malignancies (NCT01553071)^[118]^.

The SPHK1 isozyme has been extensively characterized and its diverse functions in tumor progression documented, while SPHK2 has not been as well characterized and its primary physiological functions are controversial^[44,45,57,120,121]^. Despite incomplete characterization of function and mechanism, *in vitro* and preclinical *in vivo* data document that the SPHK2-specific inhibitor ABC294640 has been shown to inhibit proliferation of tumor cells or tumors more effectively or similarly than agents that target SPHK1 in several tumor models, including ovarian^[122]^, multiple myeloma^[123]^, lung^[124]^, kidney^[58]^, breast^[58,125]^, prostate^[126]^, and pancreatic cancers^[127]^. Mechanistically, siRNA-targeted knockdown of SPHK2 expression inhibits ERK-mediated proliferation, invasion, and migration greater than knockdown of SPHK1 in kidney and breast tumor models^[122]^. In models of chemoresistant breast and ovarian cancer, ABC29460 decreases cell survival in a dose-dependent manner. Further, ABC29460 suppressed pancreatic and lung tumor growth. The proposed mechanisms for this inhibition in cell survival are inhibition of telomerase stability in lung tumor models and suppression of Myc and ribonucleotide-diphosphate reductase subunit M2 (RRM2) expression in pancreatic cancer cell lines^[122,125,127,128]^. Guan *et al*.^[124]^ demonstrated in lung cancer cells that PDPMP-mediated inhibition or knockdown of GCS potentiated ABC294640-induced antitumor activity, increased intracellular levels of ceramide, and increased apoptosis, whereas forced overexpression of GCS abrogated ABC294640 cytotoxicity against lung cancer cells. Clinically, a recently completed phase I trial ABC294640 for solid tumor patients reported acute biphasic reductions in plasma levels of S1P over 24-hour increments; however, this effect was independent of dose administered^[129]^. ABC294640 is currently being evaluated in several other clinical trials, including a phase II trial as second-line monotherapy for patients with advanced hepatocellular carcinoma (NCT02939807), a phase IIa study for treatment of patients with advanced cholangiocarcinoma (NCT03377179), and a phase Ib/II safety and efficacy study as a single agent in patients with refractory/relapsed multiple myeloma (NCT02757326).

## TAMOXIFEN AS AN INHIBITOR OF SPHINGOLIPID METABOLISM AND MODULATOR OF DRUG RESISTANCE

Tamoxifen is a standard of care drug for treatment of breast cancer, and functions as a selective estrogen receptor modulator (SERM) to competitively inhibit estradiol-estrogen receptor (ER) interaction^[130]^. Independent of ER status, tamoxifen has oncolytic activity thought to be mediated by multiple mechanisms including inhibition of sphingolipid metabolism and inhibition of the activity of the drug efflux transporter P-glycoprotein (P-gp)^[71,131,132]^. Previous literature indicated that tamoxifen enhanced the therapeutic efficacy of a wide range of agents such as paclitaxel, cisplatin, vincristine, and fenretinide in drug-resistant cancer models of colon, prostate, ovarian cancer, and neuroblastoma^[30,69,131,133,134]^. The antiproliferative effect of tamoxifen has been proposed to depend on inhibition of acid ceramidase and GCS activity, and the resulting increase in ceramide levels^[131,132]^. Devalapally *et al*.^[30]^ examined the *in vitro* and *in vivo* efficacy of encapsulated tamoxifen and paclitaxel using PEO-PCL nanoparticles in paclitaxel-resistant SKOV3. TR and wildtype SKOV3 ovarian cancer cells. As would be predicted, in tumor cells *in vitro* or tumor models *in vivo*, this formulation increased the efficiency of drug delivery, and intracellular drug retention, and increased intracellular ceramide levels and induction of apoptosis^[30]^. The data demonstrated that the combination of tamoxifen and paclitaxel decreased tumor volume and weight, induced apoptosis, and decreased GCS expression compared to control tumors. Additionally, our lab observed that the combination of tamoxifen and FTY720 inhibits proliferation of both ERα-positive and ERα-negative drug-resistant cell lines and an ERα-positive PDX model of ovarian cancer (Kreitzburg and Yoon, unpublished data). The multiple mechanisms of action of tamoxifen and its relatively high therapeutic index provide a strong rationale for combining tamoxifen with FTY720, as a strategy for treating ovarian tumors and circumventing drug resistance^[30,131,135,136]^. We suggest that therapeutics that promote ceramide accumulation by any of several pathways have broad translational potential.

## CONCLUDING REMARKS

As reviewed herein, sphingolipids, enzymes that comprise the S1P pathway, and sphingolipid metabolism have strong influence on the pathogenesis and drug-resistance in ovarian cancer. In summary, the generation and accumulation of ceramide and sphingosine is induced in response to various cellular stresses including chemotherapy, radiation, and/or oxidative stress to mediate cell death, senescence, and/or cell cycle arrest. Conversely, the metabolic conversion of ceramide to S1P, sphingomyelin, or glucosylceramide is mitogenic and inhibits antiapoptotic pathways, thereby promoting the proliferation and drug resistance of cancer cells. Because the sphingolipid metabolic pathway is implicated in multiple biological processes that are recognized to be essential for the development, progression, and drug-resistance of ovarian cancer, therapeutic modulation of sphingolipid metabolism may provide effective antitumor therapies for ovarian cancer^[13,15]^.

## Figures and Tables

**Table 1.** List of anticancer therapies targeting sphingolipid metabolism in ovarian cancer

Name	Target/activity	Stage of development	References

Ceramide analogs			
C6-ceramide nanoliposomes	Survivin, prosurvival protein kinase Cζ-dependent AKT and ERK signaling cascades, and VEGF production	Phase I	[78,81–83,94]
Inhibitors of S1P metabolism			
FTY720	S1PR1	FDA-approved for multiple sclerosis	[29,106,137]
Anti-S1P (Sphingomab)	S1P	Phase II	[43,95,96]
(Sonepcizumab)			
ABC294640	SPHK2, GCS, DES	Phase Ib and II	[122,129]
SKI-II	SPHK1, SPHK2	Preclinical	[54,138]
Tamoxifen	GCS, AC, P-gp	FDA-approved	[30]
Safingol	SPHK, PKC	Phase I	[118,139,140]

## References

[1] Romero I, Bast RC Jr. Minireview: human ovarian cancer: biology, current management, and paths to personalizing therapy. Endocrinology 2012;153:1593–602. 22416079 10.1210/en.2011-2123 PMC3320264

[2] Jayson GC, Kohn EC, Kitchener HC, Ledermann JA. Ovarian cancer. Lancet 2014;384:1376–88. 24767708 10.1016/S0140-6736(13)62146-7

[3] Eckstein N. Platinum resistance in breast and ovarian cancer cell lines. J Exp Clin Cancer Res 2011;30:91. 21967738 10.1186/1756-9966-30-91 PMC3197542

[4] Hannun YA, Obeid LM. Sphingolipids and their metabolism in physiology and disease. Nat Rev Mol Cell Biol 2018;19:175–91. 29165427 10.1038/nrm.2017.107 PMC5902181

[5] Patwardhan GA, Liu YY. Sphingolipids and expression regulation of genes in cancer. Prog Lipid Res 2011;50:104–14. 20970453 10.1016/j.plipres.2010.10.003 PMC3012148

[6] Babahosseini H, Roberts PC, Schmelz EM, Agah M. Roles of bioactive sphingolipid metabolites in ovarian cancer cell biomechanics. Conf Proc IEEE Eng Med Biol Soc 2012;2012:2436–9. 10.1109/EMBC.2012.6346456 23366417

[7] Ogretmen B. Sphingolipid metabolism in cancer signalling and therapy. Nat Rev Cancer 2018;18:33–50. 29147025 10.1038/nrc.2017.96 PMC5818153

[8] Ogretmen B, Hannun YA. Biologically active sphingolipids in cancer pathogenesis and treatment. Nat Rev Cancer 2004;4:604–16. 15286740 10.1038/nrc1411

[9] Ponnusamy S, Meyers-Needham M, Senkal CE, Saddoughi SA, Sentelle D, Selvam SP, Salas A, Ogretmen B. Sphingolipids and cancer: ceramide and sphingosine-1-phosphate in the regulation of cell death and drug resistance. Future Oncol 2010;6:1603–24. 21062159 10.2217/fon.10.116 PMC3071292

[10] Pyne NJ, Tonelli F, Lim KG, Long JS, Edwards J, Pyne S. Sphingosine 1-phosphate signalling in cancer. Biochem Soc Trans 2012;40:94–100. 22260672 10.1042/BST20110602

[11] Pyne NJ, Pyne S. Sphingosine 1-phosphate and cancer. Nat Rev Cancer 2010;10:489–503. 20555359 10.1038/nrc2875

[12] Hannun YA, Obeid LM. Principles of bioactive lipid signalling: lessons from sphingolipids. Nat Rev Mol Cell Biol 2008;9:139–50. 18216770 10.1038/nrm2329

[13] Kunkel GT, Maceyka M, Milstien S, Spiegel S. Targeting the sphingosine-1-phosphate axis in cancer, inflammation and beyond. Nat Rev Drug Discov 2013;12:688–702. 23954895 10.1038/nrd4099 PMC3908769

[14] Sentelle RD, Senkal CE, Jiang W, Ponnusamy S, Gencer S, Selvam SP, Ramshesh VK, Peterson YK, Lemasters JJ, Szulc ZM, Bielawski J, Ogretmen B. Ceramide targets autophagosomes to mitochondria and induces lethal mitophagy. Nat Chem Biol 2012;8:831–8. 22922758 10.1038/nchembio.1059 PMC3689583

[15] Morad SA, Cabot MC. Ceramide-orchestrated signalling in cancer cells. Nat Rev Cancer 2013;13:51–65. 23235911 10.1038/nrc3398

[16] Spiegel S, Milstien S. Sphingosine-1-phosphate: an enigmatic signalling lipid. Nat Rev Mol Cell Biol 2003;4:397–407. 12728273 10.1038/nrm1103

[17] Mahajan-Thakur S, Bien-Moller S, Marx S, Schroeder H, Rauch BH. Sphingosine 1-phosphate (S1P) signaling in glioblastoma multiforme-a systematic review. Int J Mol Sci 2017;18:E2448. 10.3390/ijms18112448 PMC5713415 29149079

[18] Gault CR, Obeid LM, Hannun YA. An overview of sphingolipid metabolism: from synthesis to breakdown. Adv Exp Med Biol 2010;688:1–23. 20919643 10.1007/978-1-4419-6741-1_1 PMC3069696

[19] Takabe K, Paugh SW, Milstien S, Spiegel S. “Inside-out” signaling of sphingosine-1-phosphate: therapeutic targets. Pharmacol Rev 2008;60:181–95. 18552276 10.1124/pr.107.07113 PMC2695666

[20] Liu YY, Hill RA, Li YT. Ceramide glycosylation catalyzed by glucosylceramide synthase and cancer drug resistance. Adv Cancer Res 2013;117:59–89. 23290777 10.1016/B978-0-12-394274-6.00003-0 PMC4051614

[21] Olivera A, Allende ML, Proia RL. Shaping the landscape: metabolic regulation of S1P gradients. Biochim Biophys Acta 2013;1831:193–202. 22735358 10.1016/j.bbalip.2012.06.007 PMC3484219

[22] Garcia-Barros M, Coant N, Truman JP, Snider AJ, Hannun YA. Sphingolipids in colon cancer. Biochim Biophys Acta 2014;1841:773–82. 24060581 10.1016/j.bbalip.2013.09.007 PMC3962523

[23] Park KS, Kim MK, Lee HY, Kim SD, Lee SY, Kim JM, Ryu SH, Bae YS. S1P stimulates chemotactic migration and invasion in OVCAR3 ovarian cancer cells. Biochem Biophys Res Commun 2007;356:239–44. 17349972 10.1016/j.bbrc.2007.02.112

[24] Wang D, Zhao Z, Caperell-Grant A, Yang G, Mok SC, Liu J, Bigsby RM, Xu Y. S1P differentially regulates migration of human ovarian cancer and human ovarian surface epithelial cells. Mol Cancer Ther 2008;7:1993–2002. 18645009 10.1158/1535-7163.MCT-08-0088 PMC2649755

[25] Smicun Y, Reierstad S, Wang FQ, Lee C, Fishman DA. S1P regulation of ovarian carcinoma invasiveness. Gynecol Oncol 2006;103:952–9. 16956652 10.1016/j.ygyno.2006.06.036

[26] Smicun Y, Gil O, Devine K, Fishman DA. S1P and LPA have an attachment-dependent regulatory effect on invasion of epithelial ovarian cancer cells. Gynecol Oncol 2007;107:298–309. 17716713 10.1016/j.ygyno.2007.06.024

[27] Dobbin ZC, Katre AA, Steg AD, Erickson BK, Shah MM, Alvarez RD, Conner MG, Schneider D, Chen D, Landen CN. Using heterogeneity of the patient-derived xenograft model to identify the chemoresistant population in ovarian cancer. Oncotarget 2014;5:8750–64. 25209969 10.18632/oncotarget.2373 PMC4226719

[28] Lee AJ, Roylance R, Sander J, Gorman P, Endesfelder D, Kschischo M, Jones NP, East P, Nicke B, Spassieva S, Obeid LM, Birkbak NJ, Szallasi Z, McKnight NC, Rowan AJ, Speirs V, Hanby AM, Downward J, Tooze SA, Swanton C. CERT depletion predicts chemotherapy benefit and mediates cytotoxic and polyploid-specific cancer cell death through autophagy induction. J Pathol 2012;226:482–94. 21953249 10.1002/path.2998

[29] Lee JW, Ryu JY, Yoon G, Jeon HK, Cho YJ, Choi JJ, Song SY, Do IG, Lee YY, Kim TJ, Choi CH, Kim BG, Bae DS. Sphingosine kinase 1 as a potential therapeutic target in epithelial ovarian cancer. Int J Cancer 2015;137:221–9. 25429856 10.1002/ijc.29362

[30] Devalapally H, Duan Z, Seiden MV, Amiji MM. Modulation of drug resistance in ovarian adenocarcinoma by enhancing intracellular ceramide using tamoxifen-loaded biodegradable polymeric nanoparticles. Clin Cancer Res 2008;14:3193–203. 18483388 10.1158/1078-0432.CCR-07-4973

[31] Prinetti A, Basso L, Appierto V, Villani MG, Valsecchi M, Loberto N, Prioni S, Chigorno V, Cavadini E, Formelli F, Sonnino S. Altered sphingolipid metabolism in N-(4-hydroxyphenyl)-retinamide-resistant A2780 human ovarian carcinoma cells. J Biol Chem 2003;278:5574–83. 12486134 10.1074/jbc.M207269200

[32] Swanton C, Marani M, Pardo O, Warne PH, Kelly G, Sahai E, Elustondo F, Chang J, Temple J, Ahmed AA, Brenton JD, Downward J, Nicke B. Regulators of mitotic arrest and ceramide metabolism are determinants of sensitivity to paclitaxel and other chemotherapeutic drugs. Cancer Cell 2007;11:498–512. 17560332 10.1016/j.ccr.2007.04.011

[33] Kolesnick R, Altieri D, Fuks Z. A CERTain role for ceramide in taxane-induced cell death. Cancer Cell 2007;11:473–5. 17560328 10.1016/j.ccr.2007.05.003

[34] Liu YY, Patwardhan GA, Bhinge K, Gupta V, Gu X, Jazwinski SM. Suppression of glucosylceramide synthase restores p53-dependent apoptosis in mutant p53 cancer cells. Cancer Res 2011;71:2276–85. 21278235 10.1158/0008-5472.CAN-10-3107 PMC3059346

[35] Suh DH, Kim HS, Kim B, Song YS. Metabolic orchestration between cancer cells and tumor microenvironment as a co-evolutionary source of chemoresistance in ovarian cancer: a therapeutic implication. Biochem Pharmacol 2014;92:43–54. 25168677 10.1016/j.bcp.2014.08.011

[36] Gouaze-Andersson V, Cabot MC. Glycosphingolipids and drug resistance. Biochim Biophys Acta 2006;1758:2096–103. 17010304 10.1016/j.bbamem.2006.08.012

[37] Best C, Calianese D, Szulak K, Cammarata G, Brum G, Carbone T, Still E, Higgins K, Ji F, Di W, Wanebo H, Wan Y. Paclitaxel disrupts polarized entry of membrane-permeable C6 ceramide into ovarian cancer cells resulting in synchronous induction of cell death. Oncol Lett 2013;5:1854–8. 23833655 10.3892/ol.2013.1305 PMC3701061

[38] Prinetti A, Millimaggi D, D’Ascenzo S, Clarkson M, Bettiga A, Chigorno V, Sonnino S, Pavan A, Dolo V. Lack of ceramide generation and altered sphingolipid composition are associated with drug resistance in human ovarian carcinoma cells. Biochem J 2006;395:311–8. 16356169 10.1042/BJ20051184 PMC1422777

[39] El Kaffas A, Al-Mahrouki A, Hashim A, Law N, Giles A, Czarnota GJ. Role of acid sphingomyelinase and ceramide in mechanoacoustic enhancement of tumor radiation responses. J Natl Cancer Inst 2018; doi: 10.1093/jnci/djy011. PMC6136928 29506145

[40] Oskouian B, Saba JD. Cancer treatment strategies targeting sphingolipid metabolism. Adv Exp Med Biol 2010;688:185–205. 20919655 10.1007/978-1-4419-6741-1_13 PMC3076281

[41] Dai L, Xia P, Di W. Sphingosine 1-phosphate: a potential molecular target for ovarian cancer therapy? Cancer Invest 2014;32:71–80. 24499107 10.3109/07357907.2013.876646

[42] Dai L, Liu Y, Xie L, Wu X, Qiu L, Di W. Sphingosine kinase 1/sphingosine-1-phosphate (S1P)/S1P receptor axis is involved in ovarian cancer angiogenesis. Oncotarget 2017;8:74947–61. 10.18632/oncotarget.20471 PMC5650392 29088837

[43] Schwartz BM, Hong G, Morrison BH, Wu W, Baudhuin LM, Xiao YJ, Mok SC, Xu Y. Lysophospholipids increase interleukin-8 expression in ovarian cancer cells. Gynecol Oncol 2001;81:291–300. 11330965 10.1006/gyno.2001.6124

[44] Hatoum D, Haddadi N, Lin Y, Nassif NT, McGowan EM. Mammalian sphingosine kinase (SphK) isoenzymes and isoform expression: challenges for SphK as an oncotarget. Oncotarget 2017;8:36898–929. 10.18632/oncotarget.16370 PMC5482707 28415564

[45] Maceyka M, Sankala H, Hait NC, Le Stunff H, Liu H, Toman R, Collier C, Zhang M, Satin LS, Merrill AH Jr, Milstien S, Spiegel S. SphK1 and SphK2, sphingosine kinase isoenzymes with opposing functions in sphingolipid metabolism. J Biol Chem 2005;280:37118–29. 10.1074/jbc.M502207200 16118219

[46] Siow D, Wattenberg B. The compartmentalization and translocation of the sphingosine kinases: mechanisms and functions in cell signaling and sphingolipid metabolism. Crit Rev Biochem Mol Biol 2011;46:365–75. 21864225 10.3109/10409238.2011.580097 PMC3183286

[47] Gao Y, Gao F, Chen K, Tian ML, Zhao DL. Sphingosine kinase 1 as an anticancer therapeutic target. Drug Des Devel Ther 2015;9:3239–45. 10.2147/DDDT.S83288 PMC4484649 26150697

[48] Datta A, Loo SY, Huang B, Wong L, Tan SS, Tan TZ, Lee SC, Thiery JP, Lim YC, Yong WP, Lam Y, Kumar AP, Yap CT. SPHK1 regulates proliferation and survival responses in triple-negative breast cancer. Oncotarget 2014;5:5920–33. 25153718 10.18632/oncotarget.1874 PMC4171602

[49] Pchejetski D, Bohler T, Stebbing J, Waxman J. Therapeutic potential of targeting sphingosine kinase 1 in prostate cancer. Nat Rev Urol 2011;8:569–678. 21912422 10.1038/nrurol.2011.117

[50] Hait NC, Allegood J, Maceyka M, Strub GM, Harikumar KB, Singh SK, Luo C, Marmorstein R, Kordula T, Milstien S, Spiegel S. Regulation of histone acetylation in the nucleus by sphingosine-1-phosphate. Science 2009;325:1254–7. 19729656 10.1126/science.1176709 PMC2850596

[51] Taha TA, Osta W, Kozhaya L, Bielawski J, Johnson KR, Gillanders WE, Dbaibo GS, Hannun YA, Obeid LM. Down-regulation of sphingosine kinase-1 by DNA damage: dependence on proteases and p53. J Biol Chem 2004;279:20546–54. 10.1074/jbc.M401259200 14988393

[52] Kim HS, Yoon G, Ryu JY, Cho YJ, Choi JJ, Lee YY, Kim TJ, Choi CH, Song SY, Kim BG, Bae DS, Lee JW. Sphingosine kinase 1 is a reliable prognostic factor and a novel therapeutic target for uterine cervical cancer. Oncotarget 2015;6:26746–56. 10.18632/oncotarget.4818 PMC4694949 26311741

[53] Truman JP, Garcia-Barros M, Obeid LM, Hannun YA. Evolving concepts in cancer therapy through targeting sphingolipid metabolism. Biochim Biophys Acta 2014;1841:1174–88. 24384461 10.1016/j.bbalip.2013.12.013 PMC4221100

[54] Yang YL, Ji C, Cheng L, He L, Lu CC, Wang R, Bi ZG. Sphingosine kinase-1 inhibition sensitizes curcumin-induced growth inhibition and apoptosis in ovarian cancer cells. Cancer Sci 2012;103:1538–45. 22594559 10.1111/j.1349-7006.2012.02335.x PMC7659178

[55] Beach JA, Aspuria PJ, Cheon DJ, Lawrenson K, Agadjanian H, Walsh CS, Karlan BY, Orsulic S. Sphingosine kinase 1 is required for TGF-beta mediated fibroblastto- myofibroblast differentiation in ovarian cancer. Oncotarget 2016;7:4167–82. 26716409 10.18632/oncotarget.6703 PMC4826197

[56] Neubauer HA, Pitson SM. Roles, regulation and inhibitors of sphingosine kinase 2. FEBS J 2013;280:5317–36. 23638983 10.1111/febs.12314

[57] Liu H, Toman RE, Goparaju SK, Maceyka M, Nava VE, Sankala H, Payne SG, Bektas M, Ishii I, Chun J, Milstien S, Spiegel S. Sphingosine kinase type 2 is a putative BH3-only protein that induces apoptosis. J Biol Chem 2003;278:40330–6. 10.1074/jbc.M304455200 12835323

[58] Gao P, Smith CD. Ablation of sphingosine kinase-2 inhibits tumor cell proliferation and migration. Mol Cancer Res 2011;9:1509–19. 21896638 10.1158/1541-7786.MCR-11-0336 PMC3219805

[59] Sankala HM, Hait NC, Paugh SW, Shida D, Lepine S, Elmore LW, Dent P, Milstien S, Spiegel S. Involvement of sphingosine kinase 2 in p53-independent induction of p21 by the chemotherapeutic drug doxorubicin. Cancer Res 2007;67:10466–74. 10.1158/0008-5472.CAN-07-2090 17974990

[60] Schnitzer SE, Weigert A, Zhou J, Brune B. Hypoxia enhances sphingosine kinase 2 activity and provokes sphingosine-1-phosphate-mediated chemoresistance in A549 lung cancer cells. Mol Cancer Res 2009;7:393–401. 19240180 10.1158/1541-7786.MCR-08-0156

[61] Hait NC, Avni D, Yamada A, Nagahashi M, Aoyagi T, Aoki H, Dumur CI, Zelenko Z, Gallagher EJ, Leroith D, Milstien S, Takabe K, Spiegel S. The phosphorylated prodrug FTY720 is a histone deacetylase inhibitor that reactivates ERalpha expression and enhances hormonal therapy for breast cancer. Oncogenesis 2015;4:e156. 26053034 10.1038/oncsis.2015.16 PMC4753524

[62] Kartal Yandim M, Apohan E, Baran Y. Therapeutic potential of targeting ceramide/glucosylceramide pathway in cancer. Cancer Chemother Pharmacol 2013;71:13–20. 23073611 10.1007/s00280-012-1984-x

[63] Hanada K. Intracellular trafficking of ceramide by ceramide transfer protein. Proc Jpn Acad Ser B Phys Biol Sci 2010;86:426–37. 10.2183/pjab.86.426 PMC3417804 20431265

[64] Yamaji T, Kumagai K, Tomishige N, Hanada K. Two sphingolipid transfer proteins, CERT and FAPP2: their roles in sphingolipid metabolism. IUBMB Life 2008;60:511–8. 18459163 10.1002/iub.83

[65] Lin HY, Delmas D, Vang O, Hsieh TC, Lin S, Cheng GY, Chiang HL, Chen CE, Tang HY, Crawford DR, Whang-Peng J, Hwang J, Liu LF, Wu JM. Mechanisms of ceramide-induced COX-2-dependent apoptosis in human ovarian cancer OVCAR-3 cells partially overlapped with resveratrol. J Cell Biochem 2013;114:1940–54. 23495037 10.1002/jcb.24539

[66] Rajanayake KK, Taylor WR, Isailovic D. The comparison of glycosphingolipids isolated from an epithelial ovarian cancer cell line and a nontumorigenic epithelial ovarian cell line using MALDI-MS and MALDI-MS/MS. Carbohydr Res 2016;431:6–14. 27267063 10.1016/j.carres.2016.05.006

[67] Roh JL, Kim EH, Park JY, Kim JW. Inhibition of glucosylceramide synthase sensitizes head and neck cancer to cisplatin. Mol Cancer Ther 2015;14:1907–15. 26063766 10.1158/1535-7163.MCT-15-0171

[68] Gouaze V, Liu YY, Prickett CS, Yu JY, Giuliano AE, Cabot MC. Glucosylceramide synthase blockade down-regulates P-glycoprotein and resensitizes multidrug-resistant breast cancer cells to anticancer drugs. Cancer Res 2005;65:3861–7. 15867385 10.1158/0008-5472.CAN-04-2329

[69] Liu YY, Gupta V, Patwardhan GA, Bhinge K, Zhao Y, Bao J, Mehendale H, Cabot MC, Li YT, Jazwinski SM. Glucosylceramide synthase upregulates MDR1 expression in the regulation of cancer drug resistance through cSrc and beta-catenin signaling. Mol Cancer 2010;9:145. 20540746 10.1186/1476-4598-9-145 PMC2903501

[70] Liu YY, Han TY, Giuliano AE, Cabot MC. Ceramide glycosylation potentiates cellular multidrug resistance. FASEB J 2001;15:719–30. 11259390 10.1096/fj.00-0223com

[71] Morad SA, Madigan JP, Levin JC, Abdelmageed N, Karimi R, Rosenberg DW, Kester M, Shanmugavelandy SS, Cabot MC. Tamoxifen magnifies therapeutic impact of ceramide in human colorectal cancer cells independent of p53. Biochem Pharmacol 2013;85:1057–65. 23353700 10.1016/j.bcp.2013.01.015 PMC3604153

[72] Juul N, Szallasi Z, Eklund AC, Li Q, Burrell RA, Gerlinger M, Valero V, Andreopoulou E, Esteva FJ, Symmans WF, Desmedt C, Haibe-Kains B, Sotiriou C, Pusztai L, Swanton C. Assessment of an RNA interference screen-derived mitotic and ceramide pathway metagene as a predictor of response to neoadjuvant paclitaxel for primary triple-negative breast cancer: a retrospective analysis of five clinical trials. Lancet Oncol 2010;11:358–65. 20189874 10.1016/S1470-2045(10)70018-8

[73] Lucci A, Giuliano AE, Han TY, Dinur T, Liu YY, Senchenkov A, Cabot MC. Ceramide toxicity and metabolism differ in wild-type and multidrug-resistant cancer cells. Int J Oncol 1999;15:535–40. 10427136 10.3892/ijo.15.3.535

[74] Patwardhan GA, Hosain SB, Liu DX, Khiste SK, Zhao Y, Bielawski J, Jazwinski SM, Liu YY. Ceramide modulates pre-mRNA splicing to restore the expression of wild-type tumor suppressor p53 in deletion-mutant cancer cells. Biochim Biophys Acta 2014;1841:1571–80. 25195822 10.1016/j.bbalip.2014.08.017 PMC4188706

[75] Atilla-Gokcumen GE, Bedigian AV, Sasse S, Eggert US. Inhibition of glycosphingolipid biosynthesis induces cytokinesis failure. J Am Chem Soc 2011;133:10010–3. 10.1021/ja202804b PMC3131740 21668028

[76] Liu J, Beckman BS, Foroozesh M. A review of ceramide analogs as potential anticancer agents. Future Med Chem 2013;5:1405–21. 23919551 10.4155/fmc.13.107 PMC4216473

[77] van Vlerken LE, Duan Z, Seiden MV, Amiji MM. Modulation of intracellular ceramide using polymeric nanoparticles to overcome multidrug resistance in cancer. Cancer Res 2007;67:4843–50. 17510414 10.1158/0008-5472.CAN-06-1648

[78] Overbye A, Holsaeter AM, Markus F, Skalko-Basnet N, Iversen TG, Torgersen ML, Sonstevold T, Engebraaten O, Flatmark K, Maelandsmo GM, Skotland T, Sandvig K. Ceramide-containing liposomes with doxorubicin: time and cell-dependent effect of C6 and C12 ceramide. Oncotarget 2017;8:76921–34. 29100358 10.18632/oncotarget.20217 PMC5652752

[79] Lucci A, Han TY, Liu YY, Giuliano AE, Cabot MC. Modification of ceramide metabolism increases cancer cell sensitivity to cytotoxics. Int J Oncol 1999;15:541–6. 10427137 10.3892/ijo.15.3.541

[80] Qiu L, Zhou C, Sun Y, Di W, Scheffler E, Healey S, Wanebo H, Kouttab N, Chu W, Wan Y. Paclitaxel and ceramide synergistically induce cell death with transient activation of EGFR and ERK pathway in pancreatic cancer cells. Oncol Rep 2006;16:907–13. 16969513

[81] Zhu QY, Wang Z, Ji C, Cheng L, Yang YL, Ren J, Jin YH, Wang QJ, Gu XJ, Bi ZG, Hu G, Yang Y. C6-ceramide synergistically potentiates the anti-tumor effects of histone deacetylase inhibitors via AKT dephosphorylation and alpha-tubulin hyperacetylation both in vitro and in vivo. Cell Death Dis 2011;2:e117. 21368888 10.1038/cddis.2010.96 PMC3077291

[82] Zhang X, Kitatani K, Toyoshima M, Ishibashi M, Usui T, Minato J, Egiz M, Shigeta S, Fox T, Deering T, Kester M, Yaegashi N. Ceramide nanoliposomes as a MLKL-dependent, necroptosis-inducing, chemotherapeutic reagent in ovarian cancer. Mol Cancer Ther 2018;17:50–9. 29079707 10.1158/1535-7163.MCT-17-0173 PMC5752574

[83] Devalapally H, Duan Z, Seiden MV, Amiji MM. Paclitaxel and ceramide co-administration in biodegradable polymeric nanoparticulate delivery system to overcome drug resistance in ovarian cancer. Int J Cancer 2007;121:1830–8. 17557285 10.1002/ijc.22886

[84] Stover TC, Kim YS, Lowe TL, Kester M. Thermoresponsive and biodegradable linear-dendritic nanoparticles for targeted and sustained release of a pro-apoptotic drug. Biomaterials 2008;29:359–69. 17964645 10.1016/j.biomaterials.2007.09.037

[85] Stover TC, Sharma A, Robertson GP, Kester M. Systemic delivery of liposomal short-chain ceramide limits solid tumor growth in murine models of breast adenocarcinoma. Clin Cancer Res 2005;11:3465–74. 15867249 10.1158/1078-0432.CCR-04-1770

[86] Tran MA, Smith CD, Kester M, Robertson GP. Combining nanoliposomal ceramide with sorafenib synergistically inhibits melanoma and breast cancer cell survival to decrease tumor development. Clin Cancer Res 2008;14:3571–81. 18519791 10.1158/1078-0432.CCR-07-4881

[87] Tagaram HR, Divittore NA, Barth BM, Kaiser JM, Avella D, Kimchi ET, Jiang Y, Isom HC, Kester M, Staveley-O’Carroll KF. Nanoliposomal ceramide prevents in vivo growth of hepatocellular carcinoma. Gut 2011;60:695–701. 21193455 10.1136/gut.2010.216671

[88] Liu X, Ryland L, Yang J, Liao A, Aliaga C, Watts R, Tan SF, Kaiser J, Shanmugavelandy SS, Rogers A, Loughran K, Petersen B, Yuen J, Meng F, Baab KT, Jarbadan NR, Broeg K, Zhang R, Liao J, Sayers TJ, Kester M, Loughran TP Jr. Targeting of survivin by nanoliposomal ceramide induces complete remission in a rat model of NK-LGL leukemia. Blood 2010;116:4192–201. 20671121 10.1182/blood-2010-02-271080 PMC2993625

[89] Jiang Y, DiVittore NA, Kaiser JM, Shanmugavelandy SS, Fritz JL, Heakal Y, Tagaram HR, Cheng H, Cabot MC, Staveley-O’Carroll KF, Tran MA, Fox TE, Barth BM, Kester M. Combinatorial therapies improve the therapeutic efficacy of nanoliposomal ceramide for pancreatic cancer. Cancer Biol Ther 2011;12:574–85. 21795855 10.4161/cbt.12.7.15971 PMC3218384

[90] Adiseshaiah PP, Clogston JD, McLeland CB, Rodriguez J, Potter TM, Neun BW, Skoczen SL, Shanmugavelandy SS, Kester M, Stern ST, McNeil SE. Synergistic combination therapy with nanoliposomal C6-ceramide and vinblastine is associated with autophagy dysfunction in hepatocarcinoma and colorectal cancer models. Cancer Lett 2013;337:254–65. 23664889 10.1016/j.canlet.2013.04.034 PMC3722309

[91] Ryland LK, Doshi UA, Shanmugavelandy SS, Fox TE, Aliaga C, Broeg K, Baab KT, Young M, Khan O, Haakenson JK, Jarbadan NR, Liao J, Wang HG, Feith DJ, Loughran TP Jr, Liu X, Kester M. C6-ceramide nanoliposomes target the Warburg effect in chronic lymphocytic leukemia. PLoS One 2013;8:e84648. 10.1371/journal.pone.0084648 PMC3868606 24367685

[92] Kester M, Bassler J, Fox TE, Carter CJ, Davidson JA, Parette MR. Preclinical development of a C6-ceramide nanoLiposome, a novel sphingolipid therapeutic. Biol Chem 2015;396:737–47. 25838296 10.1515/hsz-2015-0129

[93] Wang T, Feng L, Yang S, Liu Y, Zhang N. Ceramide lipid-based nanosuspension for enhanced delivery of docetaxel with synergistic antitumor efficiency. Drug Deliv 2017;24:800–10. 28502199 10.1080/10717544.2016.1225853 PMC8241063

[94] Kitatani K, Usui T, Sriraman SK, Toyoshima M, Ishibashi M, Shigeta S, Nagase S, Sakamoto M, Ogiso H, Okazaki T, Hannun YA, Torchilin VP, Yaegashi N. Ceramide limits phosphatidylinositol-3-kinase C2beta-controlled cell motility in ovarian cancer: potential of ceramide as a metastasis-suppressor lipid. Oncogene 2016;35:2801–12. 26364609 10.1038/onc.2015.330 PMC4791218

[95] O’Brien N, Jones ST, Williams DG, Cunningham HB, Moreno K, Visentin B, Gentile A, Vekich J, Shestowsky W, Hiraiwa M, Matteo R, Cavalli A, Grotjahn D, Grant M, Hansen G, Campbell MA, Sabbadini R. Production and characterization of monoclonal anti-sphingosine-1-phosphate antibodies. J Lipid Res 2009;50:2245–57. 19509417 10.1194/jlr.M900048-JLR200 PMC2759830

[96] Visentin B, Vekich JA, Sibbald BJ, Cavalli AL, Moreno KM, Matteo RG, Garland WA, Lu Y, Yu S, Hall HS, Kundra V, Mills GB, Sabbadini RA. Validation of an anti-sphingosine-1-phosphate antibody as a potential therapeutic in reducing growth, invasion, and angiogenesis in multiple tumor lineages. Cancer Cell 2006;9:225–38. 16530706 10.1016/j.ccr.2006.02.023

[97] Ader I, Gstalder C, Bouquerel P, Golzio M, Andrieu G, Zalvidea S, Richard S, Sabbadini RA, Malavaud B, Cuvillier O. Neutralizing S1P inhibits intratumoral hypoxia, induces vascular remodelling and sensitizes to chemotherapy in prostate cancer. Oncotarget 2015;6:13803–21. 25915662 10.18632/oncotarget.3144 PMC4537051

[98] Pal SK, Drabkin HA, Reeves JA, Hainsworth JD, Hazel SE, Paggiarino DA, Wojciak J, Woodnutt G, Bhatt RS. A phase 2 study of the sphingosine-1-phosphate antibody sonepcizumab in patients with metastatic renal cell carcinoma. Cancer 2017;123:576–82. 27727447 10.1002/cncr.30393

[99] Zhang L, Wang X, Bullock AJ, Callea M, Shah H, Song J, Moreno K, Visentin B, Deutschman D, Alsop DC, Atkins MB, Mier JW, Signoretti S, Bhasin M, Sabbadini RA, Bhatt RS. Anti-S1P antibody as a novel therapeutic strategy for VEGFR TKI-resistant renal cancer. Clin Cancer Res 2015;21:1925–34. 25589614 10.1158/1078-0432.CCR-14-2031 PMC4419371

[100] David OJ, Kovarik JM, Schmouder RL. Clinical pharmacokinetics of fingolimod. Clin Pharmacokinet 2012;51:15–28. 22149256 10.2165/11596550-000000000-00000

[101] Cohen JA, Barkhof F, Comi G, Hartung HP, Khatri BO, Montalban X, Pelletier J, Capra R, Gallo P, Izquierdo G, Tiel-Wilck K, de Vera A, Jin J, Stites T, Wu S, Aradhye S, Kappos L; TRANSFORMS Study Group. Oral fingolimod or intramuscular interferon for relapsing multiple sclerosis. N Engl J Med 2010;362:402–15. 20089954 10.1056/NEJMoa0907839

[102] Kappos L, Radue EW, O’Connor P, Polman C, Hohlfeld R, Calabresi P, Selmaj K, Agoropoulou C, Leyk M, Zhang-Auberson L, Burtin P, Group FS. A placebo-controlled trial of oral fingolimod in relapsing multiple sclerosis. N Engl J Med 2010;362:387–401. 20089952 10.1056/NEJMoa0909494

[103] Comi G, O’Connor P, Montalban X, Antel J, Radue EW, Karlsson G, Pohlmann H, Aradhye S, Kappos L; FTY720D2201 Study Group. Phase II study of oral fingolimod (FTY720) in multiple sclerosis: 3-year results. Mult Scler 2010;16:197–207. 20028707 10.1177/1352458509357065

[104] Patmanathan SN, Yap LF, Murray PG, Paterson IC. The antineoplastic properties of FTY720: evidence for the repurposing of fingolimod. J Cell Mol Med 2015;19:2329–40. 26171944 10.1111/jcmm.12635 PMC4594675

[105] White C, Alshaker H, Cooper C, Winkler M, Pchejetski D. The emerging role of FTY720 (Fingolimod) in cancer treatment. Oncotarget 2016;7:23106–27. 10.18632/oncotarget.7145 PMC5029614 27036015

[106] Zhang N, Qi Y, Wadham C, Wang L, Warren A, Di W, Xia P. FTY720 induces necrotic cell death and autophagy in ovarian cancer cells: a protective role of autophagy. Autophagy 2010;6:1157–67. 20935520 10.4161/auto.6.8.13614

[107] Lim KG, Tonelli F, Li Z, Lu X, Bittman R, Pyne S, Pyne NJ. FTY720 analogues as sphingosine kinase 1 inhibitors: enzyme inhibition kinetics, allosterism, proteasomal degradation, and actin rearrangement in MCF-7 breast cancer cells. J Biol Chem 2011;286:18633–40. 10.1074/jbc.M111.220756 PMC3099679 21464128

[108] Tonelli F, Lim KG, Loveridge C, Long J, Pitson SM, Tigyi G, Bittman R, Pyne S, Pyne NJ. FTY720 and (S)-FTY720 vinylphosphonate inhibit sphingosine kinase 1 and promote its proteasomal degradation in human pulmonary artery smooth muscle, breast cancer and androgen-independent prostate cancer cells. Cell Signal 2010;22:1536–42. 20570726 10.1016/j.cellsig.2010.05.022 PMC2947314

[109] Pitman MR, Woodcock JM, Lopez AF, Pitson SM. Molecular targets of FTY720 (fingolimod). Curr Mol Med 2012;12:1207–19. 22834825 10.2174/156652412803833599

[110] White C, Alshaker H, Cooper C, Winkler M, Pchejetski D. The emerging role of FTY720 (Fingolimod) in cancer treatment. Oncotarget 2016;7:23106–27. 10.18632/oncotarget.7145 PMC5029614 27036015

[111] Hisano Y, Kobayashi N, Kawahara A, Yamaguchi A, Nishi T. The sphingosine 1-phosphate transporter, SPNS2, functions as a transporter of the phosphorylated form of the immunomodulating agent FTY720. J Biol Chem 2011;286:1758–66. 21084291 10.1074/jbc.M110.171116 PMC3023470

[112] Honig SM, Fu S, Mao X, Yopp A, Gunn MD, Randolph GJ, Bromberg JS. FTY720 stimulates multidrug transporter- and cysteinyl leukotriene-dependent T cell chemotaxis to lymph nodes. J Clin Invest 2003;111:627–37. 12618517 10.1172/JCI16200 PMC151892

[113] Ishitsuka A, Fujine E, Mizutani Y, Tawada C, Kanoh H, Banno Y, Seishima M. FTY720 and cisplatin synergistically induce the death of cisplatin-resistant melanoma cells through the downregulation of the PI3K pathway and the decrease in epidermal growth factor receptor expression. Int J Mol Med 2014;34:1169–74. 25109763 10.3892/ijmm.2014.1882

[114] Li MH, Hla T, Ferrer F. FTY720 inhibits tumor growth and enhances the tumor-suppressive effect of topotecan in neuroblastoma by interfering with the sphingolipid signaling pathway. Pediatr Blood Cancer 2013;60:1418–23. 23704073 10.1002/pbc.24564 PMC3751174

[115] Marvaso G, Barone A, Amodio N, Raimondi L, Agosti V, Altomare E, Scotti V, Lombardi A, Bianco R, Bianco C, Caraglia M, Tassone P, Tagliaferri P. Sphingosine analog fingolimod (FTY720) increases radiation sensitivity of human breast cancer cells in vitro. Cancer Biol Ther 2014;15:797–805. 24657936 10.4161/cbt.28556 PMC4049795

[116] Pchejetski D, Bohler T, Brizuela L, Sauer L, Doumerc N, Golzio M, Salunkhe V, Teissie J, Malavaud B, Waxman J, Cuvillier O. FTY720 (fingolimod) sensitizes prostate cancer cells to radiotherapy by inhibition of sphingosine kinase-1. Cancer Res 2010;70:8651–61. 20959468 10.1158/0008-5472.CAN-10-1388

[117] A Safety Study of Fingolimod With Radiation and Temozolomide in Newly Diagnosed High Grade Glioma. ClinicalTrials.gov Identifier: (NCT02490930).

[118] Dickson MA, Carvajal RD, Merrill AH Jr, Gonen M, Cane LM, Schwartz GK. A phase I clinical trial of safingol in combination with cisplatin in advanced solid tumors. Clin Cancer Res 2011;17:2484–92. 21257722 10.1158/1078-0432.CCR-10-2323 PMC3078945

[119] Coward J, Ambrosini G, Musi E, Truman JP, Haimovitz-Friedman A, Allegood JC, Wang E, Merrill AH Jr, Schwartz GK. Safingol (L-threo-sphinganine) induces autophagy in solid tumor cells through inhibition of PKC and the PI3-kinase pathway. Autophagy 2009;5:184–93. 19098447 10.4161/auto.5.2.7361

[120] Pitson SM. Regulation of sphingosine kinase and sphingolipid signaling. Trends Biochem Sci 2011;36:97–107. 20870412 10.1016/j.tibs.2010.08.001

[121] Neubauer HA, Pham DH, Zebol JR, Moretti PA, Peterson AL, Leclercq TM, Chan H, Powell JA, Pitman MR, Samuel MS, Bonder CS, Creek DJ, Gliddon BL, Pitson SM. An oncogenic role for sphingosine kinase 2. Oncotarget 2016;7:64886–99. 10.18632/oncotarget.11714 PMC5323123 27588496

[122] White MD, Chan L, Antoon JW, Beckman BS. Targeting ovarian cancer and chemoresistance through selective inhibition of sphingosine kinase-2 with ABC294640. Anticancer Res 2013;33:3573–9. 24023282

[123] Venkata JK, An N, Stuart R, Costa LJ, Cai H, Coker W, Song JH, Gibbs K, Matson T, Garrett-Mayer E, Wan Z, Ogretmen B, Smith C, Kang Y. Inhibition of sphingosine kinase 2 downregulates the expression of c-Myc and Mcl-1 and induces apoptosis in multiple myeloma. Blood 2014;124:1915–25. 25122609 10.1182/blood-2014-03-559385 PMC4168346

[124] Guan S, Liu YY, Yan T, Zhou J. Inhibition of ceramide glucosylation sensitizes lung cancer cells to ABC294640, a first-in-class small molecule SphK2 inhibitor. Biochem Biophys Res Commun 2016;476:230–6. 27221045 10.1016/j.bbrc.2016.05.102

[125] Antoon JW, White MD, Meacham WD, Slaughter EM, Muir SE, Elliott S, Rhodes LV, Ashe HB, Wiese TE, Smith CD, Burow ME, Beckman BS. Antiestrogenic effects of the novel sphingosine kinase-2 inhibitor ABC294640. Endocrinology 2010;151:5124–35. 20861237 10.1210/en.2010-0420 PMC2954724

[126] Schrecengost RS, Keller SN, Schiewer MJ, Knudsen KE, Smith CD. Downregulation of critical oncogenes by the selective SK2 inhibitor ABC294640 hinders prostate cancer progression. Mol Cancer Res 2015;13:1591–601. 26271487 10.1158/1541-7786.MCR-14-0626 PMC4685021

[127] Lewis CS, Voelkel-Johnson C, Smith CD. Suppression of c-Myc and RRM2 expression in pancreatic cancer cells by the sphingosine kinase-2 inhibitor ABC294640. Oncotarget 2016;7:60181–92. 27517489 10.18632/oncotarget.11112 PMC5312377

[128] Antoon JW, Meacham WD, Bratton MR, Slaughter EM, Rhodes LV, Ashe HB, Wiese TE, Burow ME, Beckman BS. Pharmacological inhibition of sphingosine kinase isoforms alters estrogen receptor signaling in human breast cancer. J Mol Endocrinol 2011;46:205–16. 21321095 10.1530/JME-10-0116 PMC4007162

[129] Britten CD, Garrett-Mayer E, Chin SH, Shirai K, Ogretmen B, Bentz TA, Brisendine A, Anderton K, Cusack SL, Maines LW, Zhuang Y, Smith CD, Thomas MB. A phase I study of ABC294640, a first-in-class sphingosine kinase-2 inhibitor, in patients with advanced solid tumors. Clin Cancer Res 2017;23:4642–50. 28420720 10.1158/1078-0432.CCR-16-2363 PMC5559328

[130] Osborne CK. Tamoxifen in the treatment of breast cancer. N Engl J Med 1998;339:1609–18. 9828250 10.1056/NEJM199811263392207

[131] Morad SA, Cabot MC. Tamoxifen regulation of sphingolipid metabolism--therapeutic implications. Biochim Biophys Acta 2015;1851:1134–45. 25964209 10.1016/j.bbalip.2015.05.001 PMC4516673

[132] Morad SA, Levin JC, Tan SF, Fox TE, Feith DJ, Cabot MC. Novel off-target effect of tamoxifen--inhibition of acid ceramidase activity in cancer cells. Biochim Biophys Acta 2013;1831:1657–64. 23939396 10.1016/j.bbalip.2013.07.016

[133] de Vincenzo R, Scambia G, Benedetti Panici P, Bonanno G, Ercoli A, Fattorossi A, Pernisco S, Isola G, Mancuso S. Chemosensitizing effect of tamoxifen and ICI 182,780 on parental and adriamycin-resistant MCF-7 human breast cancer cells. Ann N Y Acad Sci 1996;784:517–20. 8651609 10.1111/j.1749-6632.1996.tb16273.x

[134] Ercoli A, Scambia G, De Vincenzo R, Alimonti A, Petrucci F, Fattorossi A, Isola G, Benedetti Panici P, Caroli S, Mancuso S. Tamoxifen synergizes the antiproliferative effect of cisplatin in human ovarian cancer cells: enhancement of DNA platination as a possible mechanism. Cancer Lett 1996;108:7–14. 8950203 10.1016/s0304-3835(96)04371-6

[135] Markman M, Bookman MA. Second-line treatment of ovarian cancer. Oncologist 2000;5:26–35. 10706647 10.1634/theoncologist.5-1-26

[136] Morad SA, Levin JC, Shanmugavelandy SS, Kester M, Fabrias G, Bedia C, Cabot MC. Ceramide--antiestrogen nanoliposomal combinations--novel impact of hormonal therapy in hormone-insensitive breast cancer. Mol Cancer Ther 2012;11:2352–61. 22962326 10.1158/1535-7163.MCT-12-0594 PMC3495995

[137] Zhang N, Dai L, Qi Y, Di W, Xia P. Combination of FTY720 with cisplatin exhibits antagonistic effects in ovarian cancer cells: role of autophagy. Int J Oncol 2013;42:2053–9. 23592281 10.3892/ijo.2013.1906

[138] Illuzzi G, Bernacchioni C, Aureli M, Prioni S, Frera G, Donati C, Valsecchi M, Chigorno V, Bruni P, Sonnino S, Prinetti A. Sphingosine kinase mediates resistance to the synthetic retinoid N-(4-hydroxyphenyl)retinamide in human ovarian cancer cells. J Biol Chem 2010;285:18594–602. 10.1074/jbc.M109.072801 PMC2881785 20404323

[139] Casper ES, Schwartz GK, Sugarman A, Leung D, Brennan MF. Phase I trial of dose-intense liposome-encapsulated doxorubicin in patients with advanced sarcoma. J Clin Oncol 1997;15:2111–7. 9164225 10.1200/JCO.1997.15.5.2111

[140] Schwartz GK, Ward D, Saltz L, Casper ES, Spiess T, Mullen E, Woodworth J, Venuti R, Zervos P, Storniolo AM, Kelsen DP. A pilot clinical/pharmacological study of the protein kinase C-specific inhibitor safingol alone and in combination with doxorubicin. Clin Cancer Res 1997;3:537–43. 9815717

